# A Novel Clinical and Stress Cardiac Magnetic Resonance (C-CMR-10) Score to Predict Long-Term All-Cause Mortality in Patients with Known or Suspected Chronic Coronary Syndrome

**DOI:** 10.3390/jcm9061957

**Published:** 2020-06-23

**Authors:** Victor Marcos-Garces, Jose Gavara, Jose V Monmeneu, Maria P Lopez-Lereu, Nerea Perez, Cesar Rios-Navarro, Elena De Dios, David Moratal, Gema Miñana, Julio Nuñez, Francisco J Chorro, Vicente Bodi

**Affiliations:** 1 Department of Cardiology, Hospital Clinico Universitario de Valencia, 46010 Valencia, Spain; vic_mg_cs@hotmail.com (V.M.-G.); gemineta@hotmail.com (G.M.); yulnunez@gmail.com (J.N.); francisco.j.chorro@uv.es (F.JC.); 2 Instituto de Investigacion Sanitaria INCLIVA, 46010 Valencia, Spain; Jose_4_6_90@hotmail.com (J.G.); neere_8@hotmail.com (N.P.); cesar_rios1@hotmail.com (C.R.-N.); 3 Center for Biomaterials and Tissue Engineering, Universitat Politècnica de València, 46022 Valencia, Spain; dmoratal@eln.upv.es; 4 Cardiovascular Magnetic Resonance Unit, Exploraciones Radiologicas Especiales (ERESA), 46015 Valencia, Spain; jmonmeneu@eresa.com (J.VM.); plereu@eresa.com (M.PL.-L.); 5 Centro de Investigación Biomédica en Red—Cardiovascular (CIBER-CV), 28029 Madrid, Spain; elenaddll@gmail.com; 6 Department of Medicine, Faculty of Medicine and Odontology, University of Valencia, 46010 Valencia, Spain

**Keywords:** cardiac magnetic resonance, chronic coronary syndrome, ischemic burden, prognosis, score, all-cause mortality

## Abstract

Vasodilator stress cardiac magnetic resonance (stressCMR) has shown robust diagnostic and prognostic value in patients with known or suspected chronic coronary syndrome (CCS). However, it is unknown whether integration of stressCMR with clinical variables in a simple clinical-imaging score can straightforwardly predict all-cause mortality in this population. We included 6187 patients in a large registry that underwent stressCMR for known or suspected CCS. Several clinical and stressCMR variables were collected, such as left ventricular ejection fraction (LVEF) and ischemic burden (number of segments with stress-induced perfusion defects (PD)). During a median follow-up of 5.56 years, we registered 682 (11%) all-cause deaths. The only independent predictors of all-cause mortality in multivariable analysis were age, male sex, diabetes mellitus (DM), LVEF and ischemic burden. Based on the weight of the chi-square increase at each step of the multivariable analysis, we created a simple clinical-stressCMR (C-CMR-10) score that included these variables (age ≥ 65 years = 3 points, LVEF ≤ 50% = 3 points, DM = 2 points, male sex = 1 point, and ischemic burden > 5 segments = 1 point). This 0 to 10 points C-CMR-10 score showed good performance to predict all-cause annualized mortality rate ranging from 0.29%/year (score = 0) to >4.6%/year (score ≥ 7). The goodness of the model and of the C-CMR-10 score was separately confirmed in 2 internal cohorts (*n* > 3000 each). We conclude that a novel and simple clinical-stressCMR score, which includes clinical and stressCMR variables, can provide robust prediction of the risk of long-term all-cause mortality in a population of patients with known or suspected CCS.

## 1. Introduction

Vasodilator stress cardiac magnetic resonance (stressCMR) has arisen as one of the most potent imaging techniques for diagnosis and risk assessment in patients with known or suspected chronic coronary syndrome (CCS) [1,2]. Its ability to detect inducible perfusion defects (PD) during stress first-pass perfusion can accurately predict the occurrence of obstructive coronary artery disease (CAD) [3,4], and this associates with fewer referrals for coronary angiography and revascularization with no impact on patient outcome [5,6].

Currently, CMR represents the gold-standard non-invasive imaging technique for an accurate quantification of key parameters in CCS patients such as left ventricular ejection fraction (LVEF) [7], late gadolinium enhancement (LGE) [8,9] and the presence of ischemia [10,11,12].

In the midst of a paradigm shift in health systems to deliver the most verifiable outcomes, all-cause death appears unarguable. Using all-cause death as the only end-point, we have recently reported the potential of stress-CMR for stratifying risk and guiding decision-making in CCS patients [13,14].

Beyond the value of sophisticated techniques, a thorough and individualized evaluation of clinical data must be the first and mandatory step for a correct management of diseases. In the present study, we aim to construct a combined clinical and stressCMR score to easily predict the long-term risk of all-cause mortality in a large registry of patients with known or suspected CCS.

## 2. Materials and Methods

### 2.1. Registry

Our cohort was comprised of all patients who underwent vasodilator stress CMR for known or suspected CCS in our health department from 2001 to 2016. We included 6187 patients in the final analysis. Baseline and CMR data were prospectively included in the registry, and periodic updates of the occurrence of all-cause mortality (in 2007, 2012 and 2018) were carried out.

To guarantee the robustness of data collection and avoid missing values over such a long period of time, the database was defined to include a limited number of baseline characteristics. The stressCMR studies were clinically indicated, no intervention was made, and patient clinical management was left at discretion of the cardiologists in charge. Data acquisition and analysis was performed in compliance with protocols approved by the Ethical Committee of the Hospital Clinico Universitario de Valencia (ethical approval number 2018/202). Written informed consent was obtained from all participants prior to study.

### 2.2. CMR Data Analysis

Technical aspects related to CMR studies are depicted elsewhere [13,15,16]. All studies were carried out and reported by two cardiologists accredited by the European Society of Cardiology with >10 years of experience in the use and interpretation of vasodilator stress CMR testing. In challenging cases, both operators evaluated the studies and the final results were adjudicated by consensus. Images were examined using customized software (Syngo, Siemens, Erlangen, Germany).

All patients were examined with a 1.5 T system (Sonata Magnetom, Siemens, Erlangen, Germany). After inducing vasodilation with intravenous dipyridamole (0.84 mg/kg body weight over 6 min), a gadolinium-based contrast agent was administered (dimeglumine gadopentetate or dimeglumine gadobenate at 0.1 mmol/kg or gadoteric acid at 0.15 mmol/kg). At least 3 slices in the short-axis view and 1 section in the long-axis views were acquired for hyperemia first-pass perfusion imaging using a gradient-echo sequence (inversion time: 90 ms; effective repetition time/echo time: 182 ms/1 ms; flip angle: 12°; matrix: 192 × 96; field of view: 400 × 300 mm; slice thickness: 8 mm). Ten minutes after administering the gadolinium-based contrast agent, late gadolinium enhancement imaging was performed in the same locations as in the cine images using a segmented inversion recovery steady-state free precession sequence (effective repetition time/echo time: 750 ms/1.26 ms; flip angle: 45°; matrix: 256 × 184; field of view: 340 × 235 mm; slice thickness: 7 mm). Inversion time was adjusted to nullify normal myocardium.

Left ventricular ejection fraction (LVEF, %) and left ventricular (LV) end-diastolic and end-systolic volumes indices (ml/m^2^) were quantified in cine images.

Using the 17-segment model [17], we visually defined CMR indices after the infusion of a gadolinium-based contrast agent. The evidence of segmental perfusion defects (PD) was defined as a persistent delay (in at least three consecutive temporal images in comparison with other segments in the same slice) during the first pass of contrast through the myocardium after vasodilator infusion.

The ischemic burden was defined as the number of segments showing PD post-stress. PD was disregarded in those segments exhibiting transmural LGE and in segments with non-transmural LGE in which the area with stress-induced PD did not extend beyond the area with LGE. In selected cases and when the relevance of a stress-induced PD was uncertain, resting perfusion imaging was performed after LGE.

LGE extent was visually defined as the number of segments manifesting LGE.

Inter- and intra-observer variability for all CMR indices used in the present registry can be consulted elsewhere [13].

### 2.3. Endpoint and Follow-Up

The clinical endpoint in our study was all-cause mortality, which was assigned by consensus of four cardiologists using the electronic regional health system registry. All-cause mortality updates were carried out centrally and performed in 2007, 2012 and 2018 as approved by the ethics committee. For the purpose of the present study, we used mortality data obtained in the latest revision performed from October 2018 to November 2018.

### 2.4. Statistical Analysis

We applied standard tests for assessing normal distribution of variables and to check for differences in clinical and stressCMR variables between patients according to whether or not they reached the all-cause mortality endpoint. Student T-test and Mann–Whitney U-test were used for continuous parametric and non-parametric variables, respectively. Group percentages were compared using the Chi-square test or Fisher’s exact test where appropriate. In the univariate analyses, time to all-cause mortality was assessed using Kaplan–Meier curves and log-rank test. Variables that achieved a *p* < 0.1 significance in the univariate analysis were incorporated as cofactors in a multivariable Cox proportional hazard regression model to predict time to all-cause mortality. A hierarchical model was used to avoid overfitting of variables. Hazard ratios with the corresponding 95% confidence intervals were computed. Changes in risk reclassification (using the continuous reclassification improvement index and integrated discrimination index) when stressCMR data was included in the multivariable model were computed. Receiver operating characteristic (ROC) curves to predict the all-cause mortality endpoint were computed.

Using the parameters that were independent predictors of all-cause mortality in multivariable analysis, a 10-point clinical-stressCMR risk (C-CMR-10) score was calculated. Points were assigned according to the weight of the increment in chi-square value in the multivariable Cox stepwise analysis. For the sake of simplicity and clinical applicability, and only to obtain the score, continuous variables were dichotomized using clinically meaningful cutoff points. Cut-off values have been previously validated for prognostic purposes in CAD: more than 65 years-old for age to define the elderly population, less than 50% for LVEF to define reduced ejection fraction [18] and more than 5 ischemic segments on stressCMR to define patients with extensive ischemia [13].

We performed an internal validation analysis by randomly dividing our registry in two consecutive cohorts, namely, derivation and validation cohorts. By this approach, we aimed to confirm the goodness of the multivariable model as well as the predictive power of the C-CMR-10 score (as obtained in the whole group) first in the derivation and then in the validation cohort.

Statistical significance was achieved at a two-tailed *p*-value < 0.05. The SPSS statistical package (version 15.0, SPSS Inc., Chicago, IL, USA) and STATA (version 9.0, StataCorp, College Station, TX, USA) were used throughout.

## 3. Results

### 3.1. Predictors of All-Cause Mortality: The Clinical-StressCMR Model

During a median follow-up of 5.56 years (267 weeks, range of 117–430 weeks), all-cause mortality occurred in 682 patients (11%). The baseline and CMR characteristics of the entire registry, as well as of survivors and deceased patients, are displayed in Table 1. Patients who reached the all-cause mortality endpoint were more frequently elderly, male, had diabetes mellitus and hypertension, previous history of coronary artery bypass grafting (CABG) and acute myocardial infarction and ST segment depression and T wave inversion on ECG (Table 1). Regarding stressCMR variables, deceased patients displayed more dilated LV end-systolic and end-diastolic volumes indices, more depressed LVEF and larger ischemic burden and LGE extent (Table 2).

We first constructed a clinical model only with the clinical variables and then a clinical-stressCMR model in which both clinical and stressCMR variables were combined to predict the outcome. In the clinical model, a higher age (hazard ratio (HR) 1.07 (1.06–1.08), *p* < 0.001), male sex (HR 1.61 (1.37–1.89), *p* < 0.001) and the history of diabetes mellitus (HR 1.7 (1.46–1.98), *p* < 0.001) and CABG (HR 1.39 (1.09–1.78), *p* = 0.008) were independent predictors of all-cause mortality (Table 3). By incorporating stressCMR variables in the model and thus constructing the clinical-stressCMR model, a higher age (HR 1.07 (1.06–1.08), *p* < 0.001), male sex (HR 1.36 (1.15–1.61), *p* < 0.001), the history of diabetes mellitus (HR 1.6 (1.37–1.87), *p* < 0.001), a more depressed LVEF (HR 0.98 (0.97–0.98) for increasing %, *p* < 0.001) and a more extensive ischemic burden (HR 1.04 (1.02–1.06) per segment, *p* = 0.001) were independent predictors of the all-cause mortality endpoint (Table 3).

The incorporation of stressCMR variables increased the predictive and discrimination power of the clinical model: C-statistic (clinical model) 0.689 vs. C-statistic (clinical-stressCMR model) 0.727, *p* < 0.001, net reclassification index: 0.395 (0.318–0.474), and integrated discrimination index: 0.025 (0.016–0.036). For comparative purposes, the C-statistic value of the stressCMR model alone (LVEF and ischemic burden) was 0.646.

### 3.2. C-CMR-10 Score

**Figure 1:**
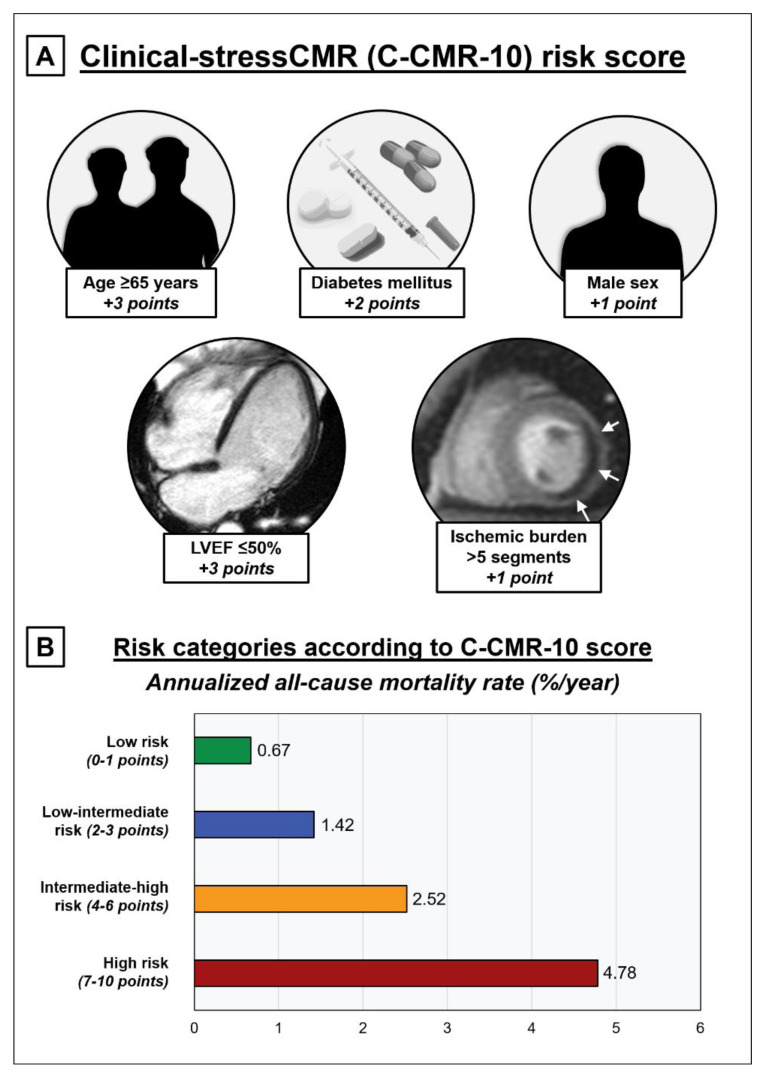
Clinical-stressCMR (C-CMR-10) score to predict all-cause mortality. (**A**) Clinical and stressCMR variables and thresholds to calculate the C-CMR-10 score. (**B**) Annualized all-cause mortality stratification according to the C-CMR-10 score risk categories. Abbreviations: LVEF = Left ventricular ejection fraction; stressCMR = Vasodilator stress cardiac magnetic resonance.

We constructed the C-CMR-10 score based on the weight of the respective increments in the global chi-square value of the model by including each independent variable in the multivariable analysis (Table 4). Continuous variables were dichotomized according to previously established criteria. Accordingly, 3 points were assigned if age > 65 years-old, 3 points if LVEF ≤ 50%, 2 points if DM, 1 point if male sex and 1 point if ischemic burden > 5 segments (Figure 1A). This score yielded a median of 4 (2–6) points and had a maximum of 10 points.

**Figure 2:**
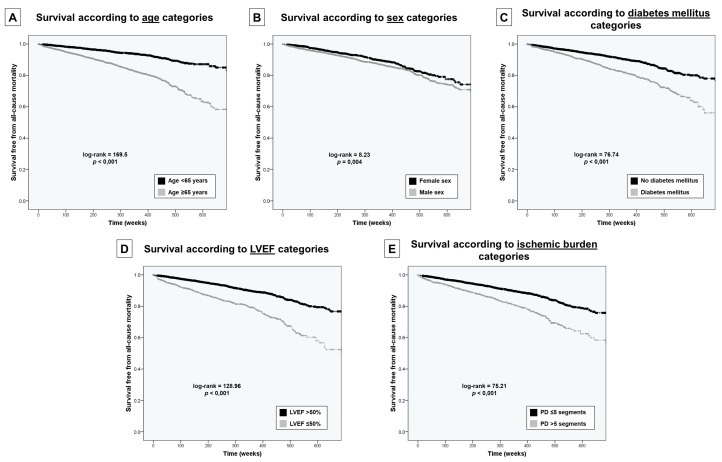
Kaplan–Meier curves to predict the risk of all-cause mortality according to the independent predictors in multivariable analysis. (**A**) Curves according to age categories (<65 and ≥65 years). (**B**) Curves according to sex categories (male and female). (**C**) Curves according to diabetes mellitus categories (with and without). (**D**) Curves according to LVEF categories (>50% and ≤50%). (**E**) Curves according to ischemic burden on stressCMR categories (≤5 and >5 segments with PD). Abbreviations: LVEF = Left ventricular ejection fraction. stressCMR = Vasodilator stress cardiac magnetic resonance. PD = Perfusion defects on stressCMR.

Variables included in the C-CMR-10 score individually predicted a decreased survival in patients falling in the “adverse” category (≥65 years-old, male sex, diabetes mellitus, LVEF ≤ 50% and >5 segments with PD) as shown in Figure 2.

### 3.3. Prediction and Stratification of All-Cause Mortality Using the C-CMR-10 Score

**Figure 3:**
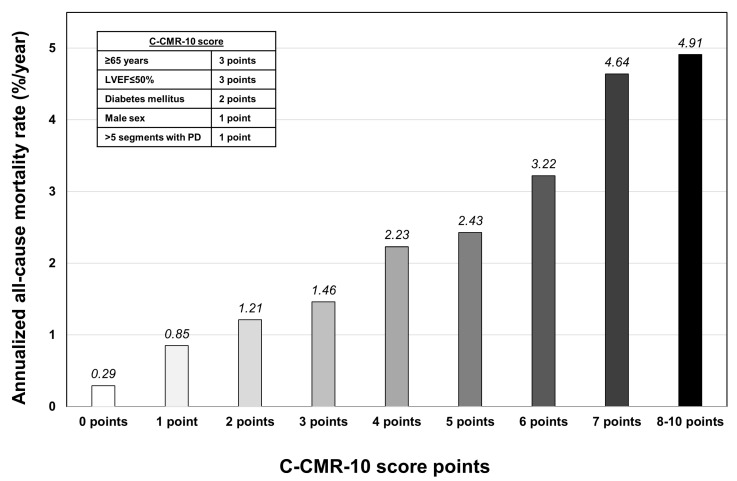
Annualized all-cause mortality risk stratification according to clinical-stressCMR (C-CMR-10) score points. Abbreviations: LVEF = Left ventricular ejection fraction. stressCMR = Vasodilator stress cardiac magnetic resonance. PD = Perfusion defects on stressCMR.

**Figure 4:**
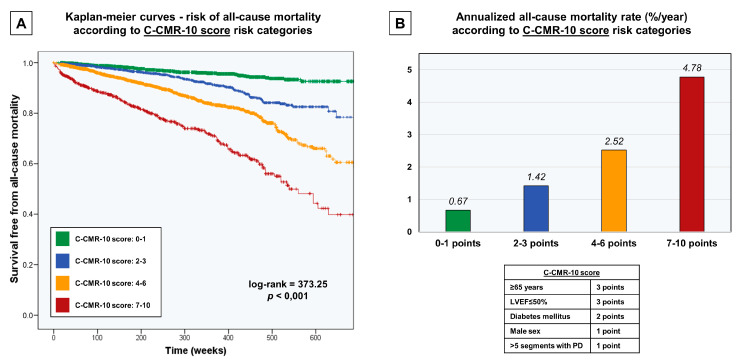
Risk stratification of all-cause mortality according to the clinical-stressCMR (C-CMR-10) score risk categories. (**A**) Kaplan–Meier curves to predict the risk of all-cause mortality during follow-up. (**B**) Annualized all-cause mortality stratification. Abbreviations: LVEF = Left ventricular ejection fraction. stressCMR = Vasodilator stress cardiac magnetic resonance. PD = Perfusion defects on stressCMR.

An increase in the number of points in the C-CMR-10 score displayed a strong linear association with a higher annualized all-cause mortality rate, ranging from a very low risk of 0.29%/year when zero points were scored to the highest risk (>4.6%/year) when ≥7 points were scored (Figure 3). We distributed the population in four risk categories according to the C-CMR-10 score: low risk (0–1 points), low-intermediate risk (2–3 points), intermediate-high risk (4–6 points) and high risk (7-10 points). This categorization permitted an intuitive stratification of the risk of all-cause mortality as derived from survival curves (Figure 4A) and annualized all-cause mortality rates (Figure 1B and Figure 4B).

### 3.4. Validation and Derivation cohorts and the C-CMR-10 Score

We carried out an internal validation of the usefulness of the C-CMR-10 score to predict all-cause mortality. For this purpose, we randomly divided our cohort in two equally sized groups: the derivation cohort (*n* = 3094) and the validation cohort (*n* = 3093). Baseline clinical and CMR characteristics of these cohorts are depicted in Supplementary Tables S1 and S2, respectively. Separate hierarchical multivariable analyses were performed in the derivation and validation cohorts using the same strategy applied for the entire study group. The independent variables included in the respective final multivariable models of the derivation and validation cohorts mirrored those included in the entire study group, namely, age, male sex, diabetes mellitus, LVEF and ischemic burden. Moreover, the weight of each variable in the derivation and validation cohorts was approximately the same weight detected in the entire group (Supplementary Table S3).

Thus, the goodness of the C-CMR-10 score for predicting all-cause mortality was separately tested in both the derivation and validation cohorts. Again, we achieved a good stratification of the occurrence of all-cause mortality in the 4 pre-defined risk categories (Supplementary Figure S1).

## 4. Discussion

The main finding of the present study is that a straightforward clinical and stressCMR (C-CMR-10) score made up of 5 clinical (age, male sex, diabetes mellitus) and stressCMR variables (LVEF and ischemic burden) permits robust stratification of the long-term all-cause mortality risk in patients with known or suspected chronic coronary syndrome.

### 4.1. Risk Stratification in CCS

Risk prediction and stratification in patients with CCS has been traditionally performed by means of routinely available clinical variables such as age, male sex, diabetes mellitus, smoking habit, hypertension, previous acute coronary syndrome or myocardial revascularization, lipid levels and history of stroke among many others [19]. Out of the myriad of clinical parameters that can exert a potential role in the prognosis of CCS patients, age, male sex and diabetes mellitus appeared as the parameters that contributed the most for the prediction of all-cause death.

LVEF has been the milestone for non-invasive risk prediction in CCS. LVEF measurement by transthoracic echocardiography is recommended in all patients with CCS, and reassessment should be performed after an acute event [20]. This recommendation is based on the different clinical management of patients with mid-range but especially reduced-LVEF, along with the fact that LVEF is a strong predictor of outcomes in patients with CCS [7] and in a broader general population [21]. Due to its higher temporal and spatial resolution and better reproducibility, CMR constitutes the gold standard for LVEF and left ventricular volumes measurement [22]. Unsurprisingly, LVEF was a relevant risk factor in our clinical-stressCMR score, underlining the importance of LVEF measurement, preferably (but not necessarily) by CMR if available, in patients with known or suspected CCS.

The role of the ischemic burden for risk stratification of CCS patients has been a matter of debate in recent years [23]. However, plenty of evidence exists supporting the deleterious effects of more extensive ischemic burden on patients’ outcomes. Two meta-analysis showed that any degree of ischemia on stressCMR was predictive of a combined major adverse cardiac events (MACE) endpoint comprised of cardiovascular (CV) death and non-fatal myocardial infarction [10,11]. Overcoming this dichotomized approach (presence vs. absence of inducible ischemia), recent research has shown that the amount of ischemia (what is called “ischemic burden”) can be used to further stratify the prognosis: The more extensive the ischemic burden, the higher the risk of adverse cardiovascular events [13,24,25,26,27].

Several studies have shown that the evidence of LGE by CMR confers an adverse prognosis to patients with CCS [8,9,28,29]. Indeed, in our study, this parameter was strongly associated with the all-cause mortality endpoint in univariate analysis but did not contribute independent information in multivariable analysis. Thus, for the sake of simplicity, we did not include LGE in the final C-CMR-10 score.

### 4.2. All-Cause Mortality as Endpoint

In CAD and CCS trials, clinical endpoints have traditionally included CV death, non-fatal myocardial infarction and unplanned coronary revascularization. Assignment of events has always been subjected to criticisms and interpretation.

The significance of individual minor endpoints (such as symptoms improvement or unplanned revascularization) can be overrated. Interpretation of the ultimate cause of death (cardiovascular or non-cardiovascular) is in many cases interpretable. In the end, this may result in the generalization of strategies that, from a statistical point of view, ameliorate combined endpoints but with neutral (or sometimes deleterious) effects on hard events.

This, along with the current situation of health systems, demands the use of accountable and unarguable endpoints. Undoubtedly, this selection limits the possibility of an in-depth analysis of the clinical course of patients. However, in our experience, interpretation of results in large registries as the one used in the present study and assignment of endpoints during long periods of time demands the use of hard events not subjected to much interpretation. Of them, the robustness of all-cause death is unquestionable.

### 4.3. Clinical Implications

Clinicians should bear in mind that patients with CCS continue to be at risk of CV events. The term “stable coronary artery disease” has been modified to “chronic coronary syndrome” in recent European Society of Cardiology guidelines [20] to emphasize the concept that CCS patients are not as “stable” as one could think [30]. Patient risk stratification in this context is of vital importance to guide clinical management in terms of secondary prevention [31] or use of invasive resources [13].

For symptomatic patients with clinical suspicion of CCS, several risk scores and complex algorithms have been proposed [32,33]. Recent guidelines recommend the routine use of clinical imaging in the management of CCS patients. As pointed out above, stressCMR represents one of the most potent non-invasive imaging techniques for diagnosis in this scenario. Nevertheless, risk scores combining clinical and stressCMR data are scarce and, in general, derived from relatively small series focused on combined endpoints.

In their study, Vincenti and colleagues examine the usefulness of stressCMR to predict a composite MACE endpoint of cardiac death, non-fatal myocardial infarction and late coronary revascularization in 1024 patients with known or suspected CCS [26]. Using four dichotomized variables, namely ischemic burden, age, LVEF and LGE, they constructed a score that permitted a good performance to predict MACE.

Several aspects have to be mentioned regarding the C-CMR 10 score obtained in our study. First, we focused on the most verifiable endpoint, i.e., all-cause mortality, which has been relatively unexplored in this scenario. Secondly, this score was derived from the largest series of patients used so far for the specific purpose of obtaining a predictive score in CCS patients that combines clinical and stressCMR data. Finally, the C-CMR-10 score permitted in a straightforward fashion predicting the annualized all-cause mortality risk by combining 5 indexes: three universally available clinical parameters (age, sex, and diabetes mellitus) and two stressCMR data (LVEF and ischemic burden).

### 4.4. Study Limitations

The registry was planned to include a large number of patients over a long period of time. In order to avoid missing values and maximize the robustness of the data collection, only a limited number of variables were defined in the database. Undoubtedly availability of additional data such as a wider inclusion of clinical variables (e.g., kidney function, previous cerebrovascular disease or peripheral artery disease), pharmacologic treatment, angiography or LVEF measurement by other techniques such as echocardiography would have permitted relevant collateral analyses now unfeasible.

As the present registry focuses on all-cause mortality as the only clinical endpoint, the usefulness of the C-CMR-10 score to predict specific cardiovascular endpoints (i.e., cardiac death or myocardial infarction) could be further explored. Moreover, the potential of the C-CMR-10 score to guide clinical management (e.g., medical therapies or a revascularization strategy) cannot be assessed with the present registry and could be addressed in subsequent studies.

Although we internally demonstrated the goodness of the C-CMR-10 in two large consecutive cohorts, further external validation would be desirable.

## 5. Conclusions

A straightforward and novel clinical-stressCMR (C-CMR-10) score, which includes clinical (age ≥ 65 years, male sex and diabetes mellitus) and stressCMR (LVEF ≤ 50% and ischemic burden > 5 segments) variables can provide robust prediction of the risk of long-term all-cause mortality in patients with known or suspected CCS. Further research should confirm the applicability of the score in daily clinical practice.

## Figures and Tables

**Table 1:** Baseline characteristics of the whole registry and of patients with and without all-cause mortality.

Variable	All Patients (*n* = 6187)	All-Cause Mortality		*p*-Value
		No (*n* = 5505)	Yes (*n* = 682)	
Age (years)	65.18 ±11.51	64.49 ± 11.55	70.77 ± 9.54	<0.001
Male sex (%)	3854 (62.3)	3395 (61.7)	459 (67.3)	0.004
DM (%)	1778 (28.7)	1497 (27.2)	281 (41.2)	<0.001
Hypertension (%)	4035 (65.2)	3544 (64.4)	491 (72)	<0.001
Hypercholesterolemia (%)	3544 (57.3)	3151 (57.2)	393 (57.6)	0.87
Current smoker (%)	1135 (18.3)	1014 (18.4)	121 (17.7)	0.714
Previous PCI (%)	1131 (18.3)	1021 (18.5)	110 (16.1)	0.128
Previous CABG (%)	419 (6.8)	345 (6.3)	74 (10.9)	<0.001
Previous infarction (%)	1165 (18.8)	1000 (18.2)	165 (24.2)	<0.001
ST-segment depression (%)	182 (2.9)	141 (2.6)	41 (6)	<0.001
T-wave inversion (%)	464 (7.5)	395 (7.2)	69 (10.1)	0.009
Left bundle branch block (%)	372 (6)	320 (5.8)	52 (7.6)	0.072
CMR-related revascularization (%) *	579 (9.4)	491 (8.9)	88 (12.9)	0.001

* Defined as those revascularization procedures by either PCI or CABG which occurred in the first 90 days after the index CMR. Abbreviations: CABG = Coronary artery bypass grafting; CMR = Cardiac magnetic resonance; DM = Diabetes mellitus; PCI = Percutaneous coronary intervention.

**Table 2:** CMR characteristics of the whole registry and of patients with and without all-cause mortality.

Variable	All Patients (*n* = 6187)	All-Cause Mortality		*p*-Value
		No (*n* = 5505)	Yes (*n* = 682)	
LVEF (%)	62.17 ± 13.63	62.85 ± 13.14	56.67 ± 16.04	<0.001
LV end-diastolic volume index (ml/m^2^)	72.76 ± 25.83	72.03 ± 24.87	78.62 ± 31.94	<0.001
LV end-systolic volume index (ml/m^2^)	29.85 ± 22.25	28.87 ± 20.92	37.81 ± 29.77	<0.001
Ischemic burden (*n* of segments with PD post-stress)	0 (0–4)	0 (0–4)	3 (0–6)	<0.001
LGE (*n* of segments)	0 (0–2)	0 (0–2)	0 (0–4)	<0.001
LGE (any degree, %)	2151 (34.8)	1826 (33.2)	325 (47.7)	<0.001

Abbreviations: CMR = Cardiac magnetic resonance; LGE = Late gadolinium enhancement; LV = Left ventricular; LVEF = Left ventricular ejection fraction; PD = Perfusion deficit.

**Table 3:** Multivariable Cox regression analysis for the all-cause mortality endpoint.

Variables	HR (95% CI)	*p*-Value
**Model 1 (Clinical)**		
Age (years) *	1.07 (1.06–1.08)	<0.001
Male sex *	1.61 (1.37–1.89)	<0.001
DM *	1.7 (1.46–1.98)	<0.001
Hypertension	1.12 (0.95–1.33)	0.189
Previous CABG *	1.39 (1.09–1.78)	0.008
Previous infarction	1.16 (0.97–1.39)	0.108
ST-segment depression	1.17 (0.85–1.62)	0.344
T-wave inversion	1.22 (0.94–1.57)	0.138
Left bundle branch block	1.31 (0.99–1.75)	0.061
CMR-related revascularization (%) ^#^	1.18 (0.93–1.49)	0.178
**Model 2 (Clinical + StressCMR)**		
Age (years)	1.07 (1.06–1.08)	<0.001
Male sex	1.36 (1.15–1.61)	<0.001
DM	1.6 (1.37–1.87)	<0.001
Previous CABG	1.13 (0.87–1.46)	0.356
LVEF (%)	0.98 (0.97–0.98)	<0.001
LV end-diastolic volume index (ml/m^2^)	1 (1–1)	0.77
LV end-systolic volume index (ml/m^2^)	1.01 (1–1.02)	0.128
Ischemic burden (*n* of segments with PD post-stress)	1.04 (1.02–1.06)	0.001
LGE (*n* of segments)	1 (0.96–1.03)	0.914
LGE (any degree, %)	1.22 (0.98–1.51)	0.071

* These variables were used to compute the Model 2 (clinical + stressCMR). ^#^ Defined as those revascularization procedures by either PCI or CABG, which occurred in the first 90 days after the index CMR. Abbreviations: CABG = Coronary artery bypass grafting; stressCMR = Vasodilator stress cardiac magnetic resonance; DM = Diabetes mellitus; LVEF = Left ventricular ejection fraction; PD = Perfusion deficit; HR (95% CI) = Hazard ratio (95% confidence intervals).

**Table 4:** Stepwise inclusion of variables in the multivariable analysis for predicting all-cause mortality. Relative weight of variables in the Model 2 (clinical + stressCMR).

	Variables	HR (95% CI)	*p*-Value	Step	Chi-Square Model	*p*-Value
Step 1	Age > 65 years	1.43 (1.35–1.52)	<0.001	175	169.41	<0.001
Step 2	Age > 65 years	1.42 (1.35–1.51)	<0.001	102.95	295.08	<0.001
	LVEF ≤ 50%	1.34 (1.27–1.42)	<0.001			
Step 3	Age > 65 years	1.4 (1.33–1.49)	<0.001	41.47	342.33	<0.001
	DM	1.67 (1.44–1.95)	<0.001			
	LVEF ≤ 50%	1.33 (1.26–1.4)	<0.001			
Step 4	Age > 65 years	1.39 (1.32–1.48)	<0.001	16.77	364.61	<0.001
	DM	1.63 (1.39–1.9)	<0.001			
	LVEF ≤ 50%	1.28 (1.21–1.36)	<0.001			
	Ischemic burden > 5 segments	1.45 (1.22–1.72)	<0.001			
Step 5	Age > 65 years	1.41 (1.34–1.5)	<0.001	11.84	377.17	<0.001
	Male sex	1.33 (1.13–1.57)	0.001			
	DM	1.64 (1.4–1.91)	<0.001			
	LVEF ≤ 50%	1.27 (1.2–1.34)	<0.001			
	Ischemic burden > 5 segments	1.42 (1.19–1.69)	<0.001			

Abbreviations: stressCMR = Vasodilator stress cardiac magnetic resonance; DM = Diabetes mellitus; LVEF = Left ventricular ejection fraction; HR (95% CI) = Hazard ratio (95% confidence intervals).

## Supplementary Materials

The following materials are available online at https://www.mdpi.com/2077-0383/9/6/1957/s1. Figure S1: Risk stratification of all-cause mortality in the derivation and validation cohorts according to the clinical-stressCMR (C-CMR-10) score risk categories. Table S1: Baseline characteristics in the derivation and validation cohorts and in patients with and without all-cause mortality. Table S2: CMR characteristics in the derivation and validation cohorts and in patients with and without all-cause mortality. Table S3: Final multivariable models for the all-cause mortality endpoint in the validation and derivation cohorts.
