# Supplementary material for: A Novel Clinical and Stress Cardiac Magnetic Resonance (C-CMR-10) Score to Predict Long-Term All-Cause Mortality in Patients with Known or Suspected Chronic Coronary Syndrome
Source: J Clin Med. 2020 Jun 23;9(6):1957. doi: 10.3390/jcm9061957 (PMC7356983; doi:10.3390/jcm9061957)
Supplement: Supplementary file 1 [file jcm-09-01957-s001.pdf]

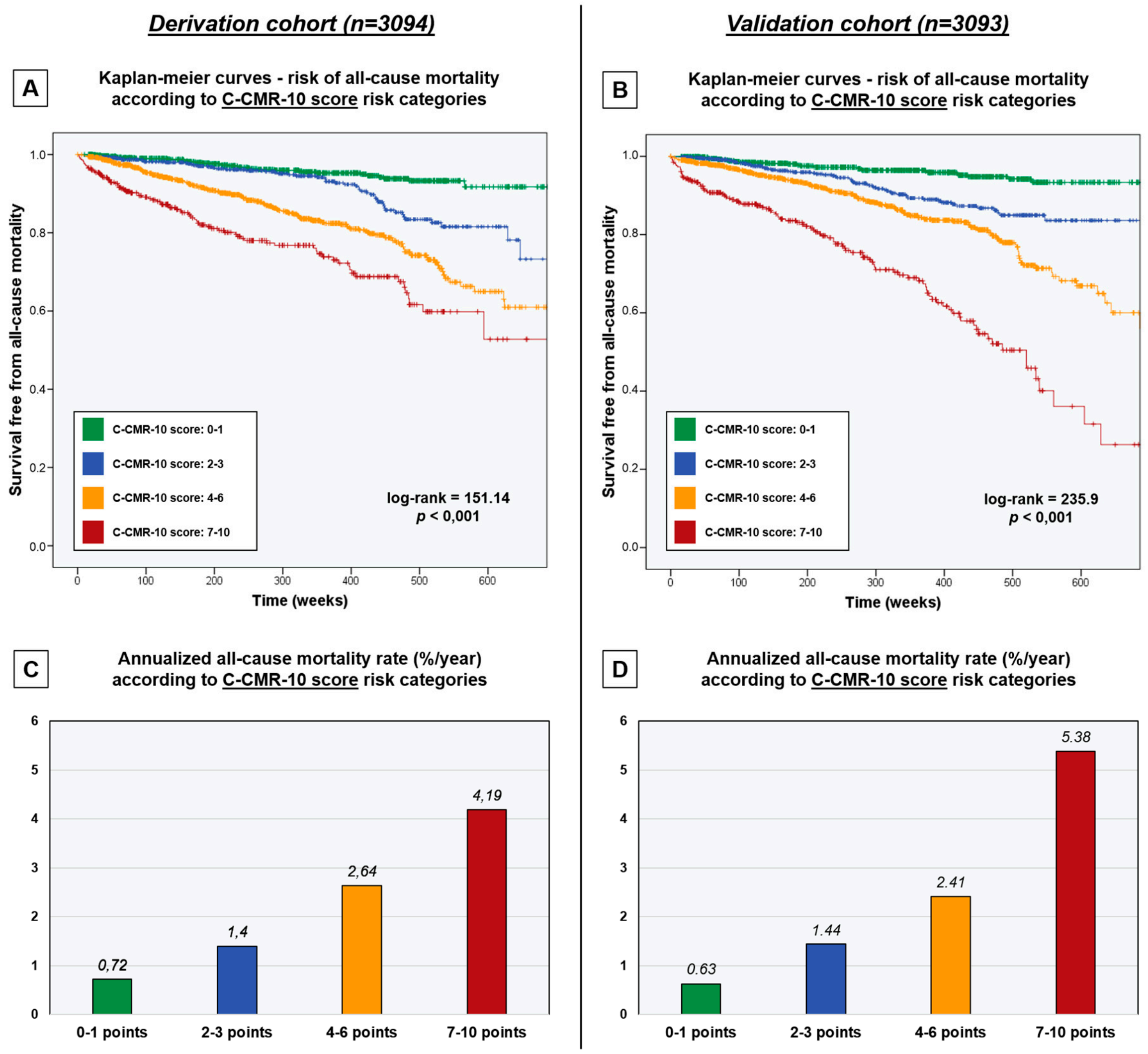

Figure S1. Risk stratification of all-cause mortality in the derivation and validation cohorts according to the clinical-stressCMR (C-CMR-10) score risk categories. stressCMR = Vasodilator stress cardiac magnetic resonance.

Table S1. Baseline characteristics in derivation and validation cohorts and in patients with and without all-cause mortality.

Variable	Derivation Cohort				Validation Cohort			
	All Patients (*n* = 3094)	All-Cause Mortality No (*n* = 2754)	All-Cause Mortality Yes (*n* = 340)	*p*-Value	All Patients (*n* = 3093)	All-Cause Mortality No (*n* = 2751)	All-Cause Mortality Yes (*n* = 342)	*p*-Value
Age (years)	65.28 ± 11.39	64.62 ± 11.44	70.63 ± 9.43	<0.001	65.07 ± 11.64	64.35 ± 11.66	70.9 ± 9.66	<0.001
Male sex (%)	1916 (61.9)	1690 (61.4)	226 (66.5)	0.076	1938 (62.7)	1705 (62)	233 (68.1)	0.028
DM (%)	890 (28.8)	740 (26.9)	150 (44.1)	<0.001	888 (28.7)	757 (27.5)	131 (38.3)	<0.001
Hypertension (%)	2014 (65.1)	1769 (64.2)	245 (72.1)	0.005	2021 (65.3)	1775 (64.5)	246 (71.9)	0.007
Hypercholesterolemia (%)	1779 (57.5)	1582 (57.4)	197 (57.9)	0.907	1765 (57.1)	1569 (57)	196 (57.3)	0.954
Current smoker (%)	559 (18.1)	501 (18.2)	58 (17.1)	0.654	576 (18.6)	513 (18.6)	63 (18.4)	1
Previous PCI (%)	556 (18)	498 (18.1)	58 (17.1)	0.708	575 (18.6)	523 (19)	52 (15.2)	0.09
Previous CABG (%)	210 (6.8)	172 (6.2)	38 (11.2)	0.001	209 (6.8)	173 (6.3)	36 (10.5)	0.006
Previous infarction (%)	572 (18.5)	483 (17.5)	89 (26.2)	<0.001	593 (19.2)	517 (18.8)	76 (22.2)	0.145

ST-segment depression (%)	84 (2.7)	65 (2.4)	19 (5.6)	0.005	98 (3.2)	76 (2.8)	22 (6.4)	0.001
T-wave inversion (%)	231 (7.5)	191 (6.9)	40 (11.8)	0.003	233 (7.5)	204 (7.4)	29 (8.5)	0.449
Left bundle branch block (%)	183 (5.9)	159 (5.8)	24 (7.1)	0.33	189 (6.1)	161 (5.9)	28 (8.2)	0.094

Abbreviations: CABG = Coronary artery bypass grafting; DM = Diabetes mellitus; PCI = Percutaneous coronary intervention.

Table S2. CMR characteristics in derivation and validation cohorts and in patients with and without all-cause mortality.

Variable	Derivation Cohort				Validation Cohort			
	All Patients (*n* = 3094)	All-Cause Mortality		*p*-Value	All Patients (*n* = 3093)	All-Cause Mortality		*p*-Value
		No (*n* = 2754)	Yes (*n* = 340)			No (*n* = 2751)	Yes (*n* = 342)	
LVEF (%)	62.14 ± 13.6	62.73 ± 13.14	57.37 ± 16.06	<0.001	62.2 ± 13.66	62.97 ± 13.14	55.98 ± 16.02	<0.001
LV end-diastolic volume index (mL/m²)	72.47 ± 25.55	71.84 ± 24.65	77.59 ± 31.45	0.001	73.05 ± 26.1	72.23 ± 25.09	79.64 ± 32.43	<0.001
LV end-systolic volume index (mL/m²)	29.83 ± 22.31	28.93 ± 20.93	37.14 ± 30.38	<0.001	29.87 ± 22.19	28.8 ± 20.92	38.47 ± 29.18	<0.001
Ischemic burden (*n* of segments with PD post-stress)	0 (0–4)	0 (0–4)	3 (0–6)	<0.001	0 (0–4)	0 (0–4)	3 (0–6)	<0.001
LGE (*n* of segments)	0 (0–2)	0 (0–2)	0 (0–4)	<0.001	0 (0–2)	0 (0–2)	0 (0–4)	<0.001

Abbreviations: CMR = Cardiac magnetic resonance; LGE = Late gadolinium enhancement; LV = Left ventricular; LVEF = Left ventricular ejection fraction; PD = Perfusion deficit.

Table S3. Final multivariable models for the all-cause mortality endpoint in the derivation and validation cohorts.

Variables	HR (95% CI)	*p*-Value
**Derivation Cohort**		
Age (years)	1.06 (1.06–1.08)	<0.001
Male sex	1.41 (1.12–1.79)	0.004
DM	1.76 (1.42–2.19)	<0.001
LVEF (%)	0.98 (0.98–0.99)	<0.001
Ischemic burden (*n* of segments with PD post-stress)	1.04 (1.01–1.07)	0.005
**Validation Cohort**		
Age (years)	1.07 (1.06–1.08)	<0.001
Male sex	1.32 (1.04–1.67)	0.023
DM	1.47 (1.18–1.83)	0.001
LVEF (%)	0.97 (0.97–0.98)	<0.001
Ischemic burden (*n* of segments with PD post-stress)	1.03 (1–1.06)	0.034

Abbreviations: DM = Diabetes mellitus; LVEF = Left ventricular ejection fraction; PD = Perfusion deficit.
